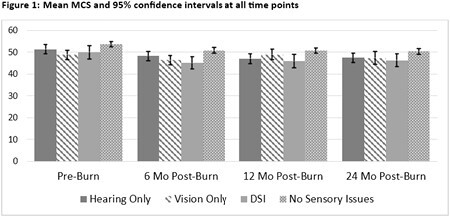# 36 The Impact of Sensory Issues on Health-related Quality of Life for People with Burn Injuries

**DOI:** 10.1093/jbcr/irae036.036

**Published:** 2024-04-17

**Authors:** Geun-woo Oh, Caitlin M Orton, Barclay T Stewart, Jeffrey C Schneider, Haig A Yenikomshian, Xinyao deGrauw, Maiya I Pacleb

**Affiliations:** Northwest Regional Burn Model System, Seattle, Washington; UW Medicine Regional Burn Center, Harborview Medical Center, Seattle, WA; University of Washington, Seattle, WA; Spaulding Rehabilitation Hospital/Harvard Medical School, Boston, MA; University of Southern California, Los Angeles, CA; Northwest Regional Burn Model System, Seattle, Washington; UW Medicine Regional Burn Center, Harborview Medical Center, Seattle, WA; University of Washington, Seattle, WA; Spaulding Rehabilitation Hospital/Harvard Medical School, Boston, MA; University of Southern California, Los Angeles, CA; Northwest Regional Burn Model System, Seattle, Washington; UW Medicine Regional Burn Center, Harborview Medical Center, Seattle, WA; University of Washington, Seattle, WA; Spaulding Rehabilitation Hospital/Harvard Medical School, Boston, MA; University of Southern California, Los Angeles, CA; Northwest Regional Burn Model System, Seattle, Washington; UW Medicine Regional Burn Center, Harborview Medical Center, Seattle, WA; University of Washington, Seattle, WA; Spaulding Rehabilitation Hospital/Harvard Medical School, Boston, MA; University of Southern California, Los Angeles, CA; Northwest Regional Burn Model System, Seattle, Washington; UW Medicine Regional Burn Center, Harborview Medical Center, Seattle, WA; University of Washington, Seattle, WA; Spaulding Rehabilitation Hospital/Harvard Medical School, Boston, MA; University of Southern California, Los Angeles, CA; Northwest Regional Burn Model System, Seattle, Washington; UW Medicine Regional Burn Center, Harborview Medical Center, Seattle, WA; University of Washington, Seattle, WA; Spaulding Rehabilitation Hospital/Harvard Medical School, Boston, MA; University of Southern California, Los Angeles, CA; Northwest Regional Burn Model System, Seattle, Washington; UW Medicine Regional Burn Center, Harborview Medical Center, Seattle, WA; University of Washington, Seattle, WA; Spaulding Rehabilitation Hospital/Harvard Medical School, Boston, MA; University of Southern California, Los Angeles, CA

## Abstract

**Introduction:**

The association between sensory issues with lowered health-related quality of life (HRQOL) has been repeatedly described, mainly in the elderly population with age-related hearing and vision issues. Although sensory issues after major burn injuries are common, the relationship between these new impairments and HRQOL after burn injury is unknown. We aimed to understand the impact of sensory issues on HRQOL to inform care and rehabilitation paradigms.

**Methods:**

Data from a multicenter national longitudinal database of people living with burn injuries were analyzed. Participant data were stratified into self-report (yes/no) of currently experiencing hearing loss, vision problems, dual sensory impairment (DSI), or no issues at the time of data collection (discharge, 6-, 12-, and 24-months post-injury). The primary outcome, patient-reported VR-12/PROMIS Global-10 physical component scores (PCS) and mental component scores (MCS), were collected at discharge (pre-injury recall) and all follow-up time points. Mixed-effect linear regressions were used to assess the impact of sensory issues on MCS and PCS at each time point.

**Results:**

The study included 1,113 participants. Participants with sensory issues had significantly lower MCS and PCS scores than those without sensory issues at pre-injury (p < 0.05, Figures 1 and 2). All cohorts experienced a significant PCS loss at 6-, 12-, and 24-months post-injury compared to pre-injury scores (p < 0.001). All cohorts experienced a significant MCS loss at 6 months post-injury compared to pre-injury (p < 0.05). The hearing-only and no sensory impairments cohorts continued to experience a significant MCS loss at 12- and 24-month post-injury (p < 0.05). DSI cohort continued to experience a significant MCS loss at 12- months (p < 0.05). The vision-only cohort had more PCS loss than the no sensory impairment cohort at 24-months (-3.3, p< 0.05).

**Conclusions:**

Although there were no statistically significant losses of participants with sensory impairments compared to those with no sensory issues, these cohorts reported consistently lower scores overall during the 24 months post-injury. Given these lower HRQOL scores, systematic screening and focused management of sensory impairments during and after rehabilitation from a burn injury should be considered.

**Applicability of Research to Practice:**

Integration of specialized mental and physical health burn rehabilitation programs that address person-specific needs may improve short- and long-term outcomes for people with hearing problems, vision issues, or DSI.